# Percutaneous Device to Narrow the Coronary Sinus: Shifting Paradigm in the Treatment of Refractory Angina? A Review of the Literature

**DOI:** 10.3389/fcvm.2016.00042

**Published:** 2016-10-21

**Authors:** Daniela Benedetto, Masieh Abawi, Pieter R. Stella, Freek Nijhoff, Maxime D. M. Lakemeier, Friso Kortlandt, Pieter A. Doevendans, Pierfrancesco Agostoni

**Affiliations:** ^1^University Medical Centre Utrecht, Utrecht, Netherlands; ^2^University of Milan, Milan, Italy; ^3^St. Antonius Hospital, Nieuwegein, Netherlands

**Keywords:** refractory angina, sinus coronarius reducer

## Abstract

Refractory angina pectoris is defined as a chronic debilitating condition characterized by the presence of chronic anginal symptoms due to a severe obstructive and/or diffuse coronary artery disease that cannot be controlled by the combination of medical therapy and/or revascularization (percutaneous or surgical). In addition, the presence of myocardial ischemia as a cause of the symptoms must have been documented. The coronary sinus reducer (CSR) is a recently introduced percutaneous device to treat patients with severe anginal symptoms refractory to optimal medical therapy and not amenable to conventional revascularization. The purpose of this review is to describe the current evidence from available studies measuring the clinical effect of the CSR implantation on the health and well-being of patients with refractory angina.

## Introduction

Refractory angina pectoris is defined as a chronic debilitating condition characterized by the presence of anginal symptoms due to a severe obstructive and/or diffuse coronary artery disease (CAD) that cannot be controlled by the combination of medical therapy and/or revascularization (percutaneous or surgical).

Despite the numerous methods of treatment of CAD, a growing number of patients (10–15%) with severe chronic ischemic heart disease continue to have refractory angina not susceptible to therapeutic alternatives (1). These patients are often labeled “end-stage” or “no-option” patients and represent a complex and heterogeneous population (2, 3). The factors responsible for the heterogeneity of these patients are the severity and frequency of angina [measured with the class of angina of the Canadian Cardiovascular Society (CCS)], the extent of CAD, the presence of recurrent coronary restenosis after percutaneous treatment, the presence of an occluded bypass graft in post-coronary artery bypass graft (CABG) surgery patients, and the presence of coronary chronic total occlusions. These may explain the difficulties encountered in assessing the effectiveness and mechanisms of action of various proposed therapeutic alternatives (4–6).

There are limited data regarding the natural history and predictors of mortality for patients with refractory angina (7). A retrospective study of the Cleveland Clinic in 500 consecutive patients undergoing cardiac catheterization showed that 59 patients had ischemia but were not susceptible to revascularization (8). The 1-year mortality in this small cohort of patients was 17% and has led many to believe that patients with refractory angina are at high risk of mortality (8). A recent study at Duke University investigated a database of 1908 consecutive patients with refractory angina who underwent catheterization (7). The analysis of mortality rates has shown that patients with clinically stable CAD who were under optimal medical therapy had similar mortality but a high incidence of re-hospitalization over the next 3 years of follow-up (7, 9). Moreover, patients with refractory angina use many anti-ischemic medications, experience impaired quality of life because of debilitating symptoms, and are relatively young (60–65 years) (10). These results indicate the need for new therapies targeting the reduction of symptoms in this population, taking into account also the potential impact in terms of health-care consumption and cost (6). Several anti-ischemic therapies (e.g., novel medical treatment such as ivabradine and ranolazine, extracorporeal shockwave therapy, spinal cord stimulation, transmyocardial laser revascularization, gene therapy, and cell therapy) have been investigated, none of which has become mainstream (5, 11). Currently, treatment options for this patient group are limited mainly to traditional anti-anginal therapy and to modification of risk factors (12, 13).

Animal studies have revealed that the increase of venous pressure at the level of the coronary sinus (CS) is able to reduce myocardial ischemia leading to a redistribution of blood from the non-ischemic to the ischemic territories (14–17). Recently, the coronary sinus reducer (CSR) has been introduced in clinical practice as a new device-based treatment for patients with refractory angina (18, 19). Implantation of the CSR is the percutaneous equivalent of surgical CS narrowing as described by Beck et al. (20, 21) and shows promise as treatment for refractory angina. Although Beck-II procedure has little in common with the CSR as the first is a surgical operation, meanwhile the second is a percutaneous intervention; however, the concept of narrowing the CS is similar, and this makes the two procedures conceptually comparable (interestingly both procedures aim at a residual lumen diameter in the CS around 3 mm).

## Device and Implantation Procedure

**Figure 1:**
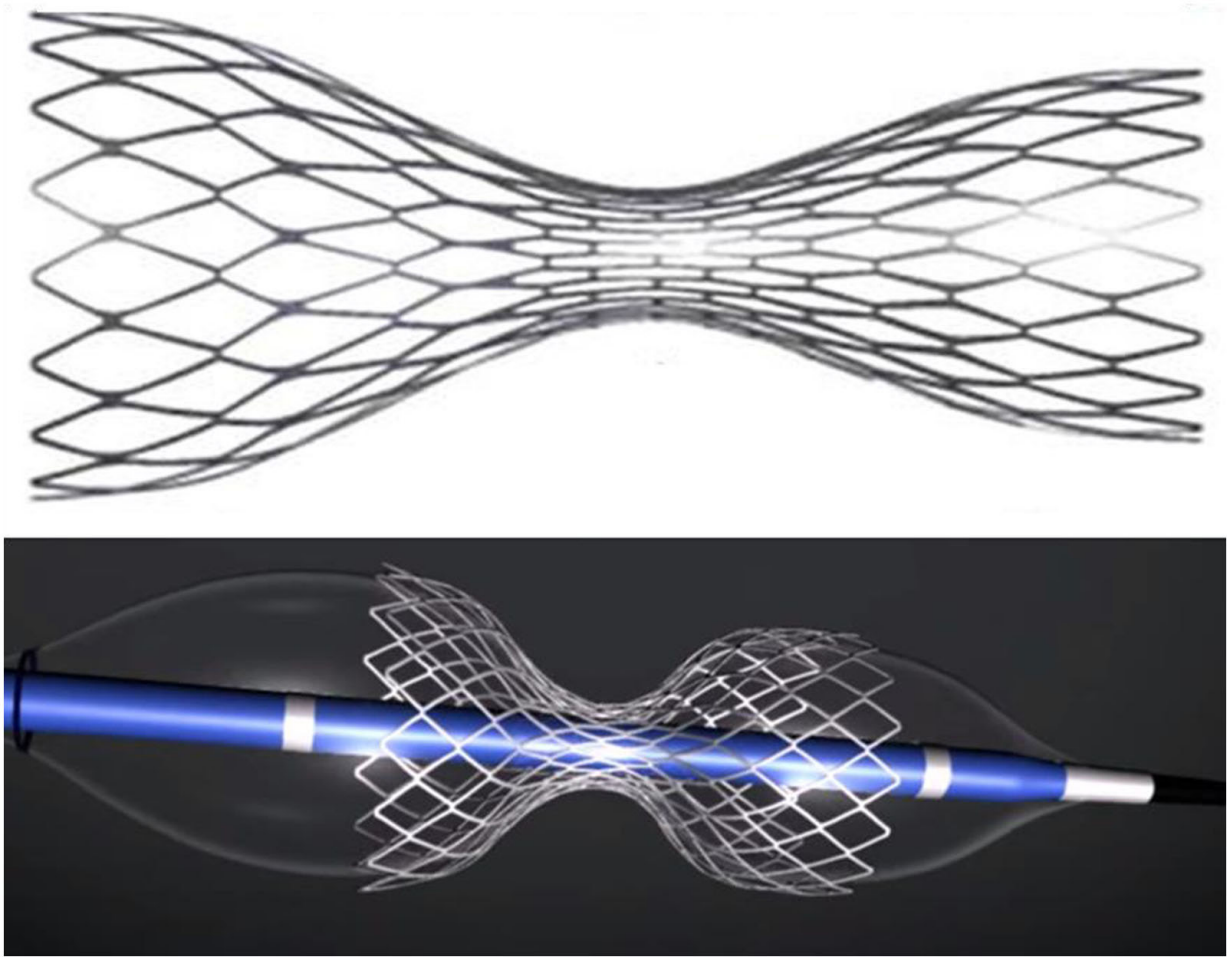
**The reducer device**. Above – expanded device. Below – expanded device mounted on the delivery balloon with their hourglass shape.

The CSR is an endoluminal device, which consists of a stainless steel stent in the shape of an hourglass mounted on an expandable balloon with the same shape once inflated (Figure 1). The device is intended to be implanted percutaneously into the CS to create a controlled narrowing of the CS. The CS is the final step in the cardiac venous drainage; thus, a controlled stenosis at this level can lead to a slight increase of the coronary venous pressure. As demonstrated in a preclinical study, the mean pressure gradient assessed across the reducer was 3.71 ± 1.75 mmHg immediately after implantation (18). The device is provided in a single model (“one-size-fits-all” design) and is suitable for a wide range of anatomies of the CS (from a minimum diameter of 9 mm to a maximum of 14 mm). The device received CE approval in November 2011 for the treatment of refractory angina.

**Figure 2:**
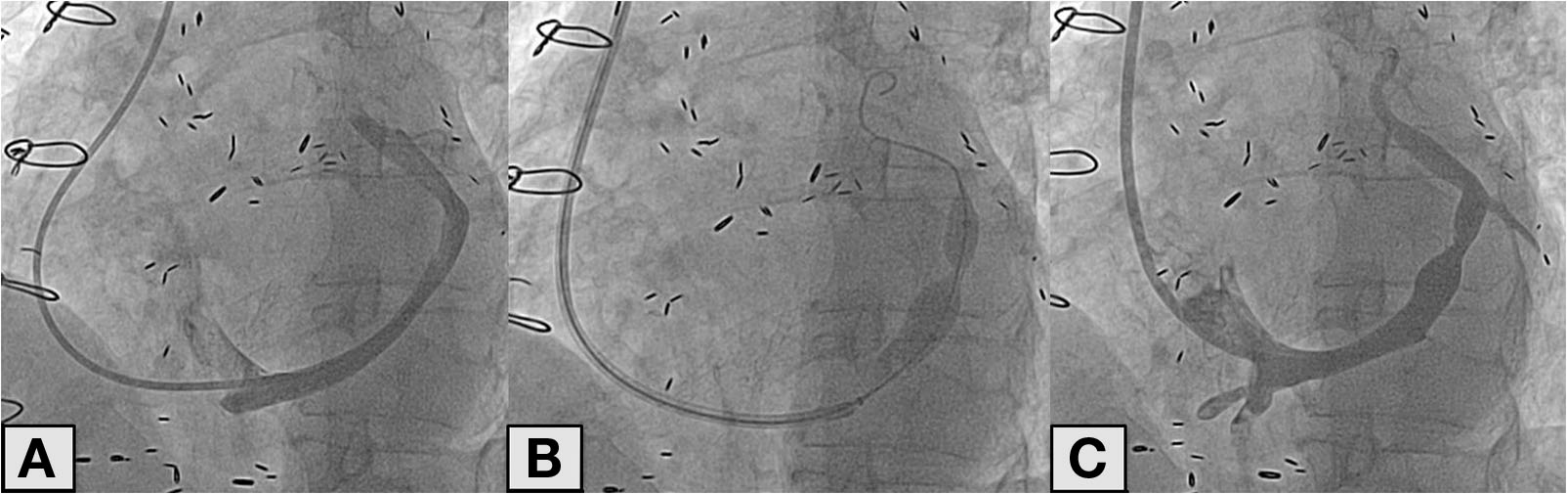
**Angiographic image of the coronary sinus (A)**. Deployment of the reducer in the coronary sinus **(B)**. Final angiographic control after reducer implantation **(C)**.

The implantation is performed under fluoroscopic guidance through percutaneous approach from the right-jugular vein under local anesthesia and through venapuncture. This approach is more practicle than femoral approach because *via* the latter, the sharp bend from the inferior vena cava to the coronary sinus would be much more difficult to tackle than the bend from the superior vena cava to the coronary sinus from the jugular vein. Furthermore, a 6-Fr diagnostic catheter (Multipurpose or Amplatz left) is used to measure the right ventricular pressure (which must be less than 15 mmHg, to avoid treating patients with uncontrolled heart failure) and to selectively cannulate the CS. Subsequently to contrast injection to assess whether the CS is suitable for implantation, a 9-Fr guiding catheter is advanced in the distal CS with the support of the 6-Fr diagnostic catheter (“mother-and-child” technique). Once the 9-Fr guiding catheter is positioned as distally as possible in the CS, the 6-Fr diagnostic catheter is retracted and the reducer is advanced inside the 9-Fr guiding up to the place chosen for the implant. The 9-Fr guiding catheter is then retracted in order to expose the reducer. The next step is the inflation of the balloon on which the reducer is mounted; the pressure ranges from 2 to 6 atm depend on the diameter of the CS. An injection of additional contrast during inflation of the reducer helps evaluate a possible slight oversizing of the reducer as compared to the diameter of the CS. After deflation, the balloon is gently retrieved in order to avoid the displacement of the reducer. A final angiogram is then performed to evaluate the correct position of the device and the absence of any complications such as perforations (Figure 2). The procedure is performed in an outpatient setting. Patients are discharged after a few hours, upon confirmation that the access site (jugular vein) is properly closed. After the procedure, dual antiplatelet therapy with aspirin and clopidogrel is recommended for a month (as for bare metal stents).

## Safety and Efficacy Performances

In various studies, the CSR was safely implanted percutaneously *via* the jugular vein, and it was associated with an improvement in anginal symptoms and also in the parameters of ischemia, in patients with refractory angina, who were not candidates for any form of revascularization (18, 22–25). These results support further evaluation of the CSR as an alternative treatment for patients with chronic refractory angina, who are not candidates for coronary revascularization.

The first study was a multicenter, non-randomized, prospective, and first-in-man study, which revealed that the use of the CSR in patients with refractory angina was safe and feasible (18). In particular, it involved 15 patients with refractory angina treated with the device. The installation of all devices was completed successfully. No procedural adverse events occurred. Most of the patients (85%) significantly improved their anginal symptoms, and also, the extent and severity of myocardial ischemia detected by dobutamine echocardiography significantly reduced in 8 out of 13 patients (18). Also, there was a medically significant reduction in extent and severity of myocardial ischemia in 4 out of 10 patients detected by Thallium SPECT studies (18). Clinical improvement was reported by the patients a few weeks after the procedure and not immediately after implantation. These results support the hypothesis that the controlled diameter reduction of the CS accompanied by an increase in venous pressure may improve myocardial perfusion during stress with consequent reduction of ischemia (18).

Another study evaluated the clinical results during 6 months of follow-up among 21 patients treated with the reducer (23). The results came from two centers (Antwerp Cardiovascular Center, Antwerp, Belgium, and Tel Aviv Medical Center, Tel Aviv, Israel), where the device is currently used to treat patients with refractory angina, with objective evidence of myocardial ischemia and ejection fraction ≥25%, who were not candidates for surgical or percutaneous coronary revascularization. The results demonstrate that the implant of the CSR was associated with a significant improvement in angina class (CCS class decreased significantly 6 months after the implant from an average of 3.3 ± 0.6 to 2.0 ± 1.0). In 85% of the cases, the improvement in functional CCS class was significantly observed. Clinical improvement was reported starting a few weeks after the procedure and maintained at follow-up.

The third study is the recently published multicenter randomized COronary SInus Reducer for treatment of refractory Angina (COSIRA) sham-controlled trial, which confirmed the efficacy of CS narrowing by reducer implantation as treatment for refractory angina (24). The study was conducted in 11 clinical centers and enrolled 104 patients. All patients underwent catheterization for angiography of the CS, and once confirmed that the vessel was treatable with the reducer, patients were randomization. Half of the patients underwent CSR implantation; the other half was not treated and formed the sham-control group. Dual antiplatelet therapy was given for at least 1 week before the procedure and for 6 months after the procedure in the two study groups. Care was taken to maintain patients blinded to the treatment. In addition, the clinical evaluation of patients before and after the treatment was performed by cardiologists not involved with the procedure and not informed about the treatment received by the patients themselves. So, the study can be called double-blind because patients and physicians that assessed the clinical status of the patients did not know the therapy performed during the procedure. A total of 18 out of 52 patients in the treatment group and 8 out of 52 in the control group had an improvement of at least 2 CCS classes, which was the primary endpoint of the study (24). Thus, a significant improvement in at least two CCS classes (35 versus 15%, *p* = 0.02) was noted with the CSR. Moreover, in the treatment group, 71% of patients (37 of 52 patients) had an improvement of at least one CCS class, compared to 42% (22 of 52) in the control group (*p* = 0.003). In addition, quality of life was improved in the CSR group as compared to the control group. No significant differences were observed between the two groups with respect to changes in the secondary endpoints, such as the duration of exercise and time to ST segment changes during ergometry, or the variation of the indices of ischemia by stress echocardiography or myocardial scintigraphy (24).

From 2014, the CSR stent is regularly used in our institution as a treatment option in clinical practice for the treatment of refractory angina. In 2014, 23 patients have been treated and a retrospective registry has been set up to assess the results of the implant of the reducer in these real-world patients (25). Patients were included based on the following criteria: (1) symptomatic angina CCS classes 2–4, despite optimal medical therapy; (2) evidence of inducible myocardial ischemia, caused by at least one of the following tests – cardiovascular cycle ergometer exercise test, myocardial scintigraphy, stress echocardiogram, or stress magnetic resonance imaging; (3) CAD not suitable for revascularization according to the decision of the Heart team. Unlike the COSIRA (24) in which only patients were included with a positive dobutamine stress echocardiography, in this registry, all forms of proven inducible ischemia have been accepted for the inclusion of patients. In line with previous studies (18, 24), the CSR implantation in these real-world patients was safe with no procedural-related complications. The majority of patients (74%) experienced a significant reduction in anginal symptoms few weeks following the procedure (25).

Theoretically, several complications may occur during or after the CSR implantation (CS dissection during catheter or wire manipulation, direct or late device migration, cardiac tamponade due to CS rapture); however, up to now, there are no data exist regarding the complications. Up to now, no cases of device occlusion has been shown during follow-up in all the patients. Also, no atrial arrhythmias have been described. The atrium does not get the influence of the device in terms of increased pressure, indeed the increase in pressure occurs only in the coronary vascular tree and that seems to be the mechanism of action.

## Mechanism of Action

The suggested anti-ischemic effect of the CSR is based on the hypothesis first described by Camici et al. (2), and the first clinical results of the CSR implantation in human were published by Banai et al. (18). In a normal heart, during exercise, there is a sympathetic-mediated vasoconstriction of the sub-epicardial vessels, which leads to an increase of blood flow to the sub-endocardial capillaries (26). In patients with obstructive CAD, this physiological compensation mechanism is dysfunctional (27). In addition, in the presence of ischemia, altered contractility and the high left ventricular end-diastolic pressure exert an external pressure on the sub-endocardial capillaries, which further increases the flow resistance toward the sub-endocardium, leading to a vicious circle that determines a worsening of sub-endocardial ischemia. High pressure upstream of the CS determines an increase of the pressure in the capillaries and venules, which will result in a slight expansion of the diameter of the capillaries and a significant reduction of the flow resistance. The resulting reduction in sub-endocardial capillary resistance subsequent to reducer implantation involves the rebalancing of the ratio between sub-epicardial and sub-endocardial blood flow that is pathologically impaired in ischemic myocardium (24). The result of this process is thus an improvement in the flow of blood to the sub-endocardial ischemic layers (28). This effect appears to take action in the first few months after reducer implantation as the stenosis becomes effective only once the device is covered with neointimal tissue, and this endothelialization process occurs in a few months (18, 24). Interestingly, porcine studies demonstrated that the pressure gradient across the reducer device decreases during 6 months follow-up (18).

The second potential mechanism of action is linked to the possible angiogenic effect of the device (29). In the early 90s, scientific research introduced new treatments for retrograde reperfusion of acutely ischemic myocardium through a kind of small balloon pump inserted into the CS, assuming that this device could reduce the post-infarction damage increasing venous pressure downstream of the ischemic territory (17). Histological findings of these studies suggest the possible intra-myocardial and epicardial angiogenesis due to proliferation of small- and medium-sized arteries that constitute the collateral circulation of the ischemic territory. However, these findings are still debated (29). Moreover, despite the careful selection of patients, the results may be influenced by a placebo effect because of the subjective nature of the major endpoint assessed. Future studies with more patients and long-term follow-up are needed to explore any possible objective effect of the CSR.

## The CS Reducer as a Treatment Option for Refractory Angina

The development of new therapies for patients with refractory angina should focus not only on reducing the risk of death and myocardial infarction but also (and perhaps especially) on alleviation of angina and improvement of quality of life. In fact, the prevalence of refractory angina continues to increase in relation to the increase in survival of patients with ischemic heart disease and the aging of the population. Although data on the incidence of refractory angina are scarce, in about 10–15% of the cases, patients with stable angina and evidence of ischemia are not amenable to percutaneous or surgical revascularization (1). These patients are mainly debilitated by poor quality of life and a high level of depression and anxiety because of their disease (30). Recent studies have highlighted the need to improve the quality of life of these patients, even more as there is growing evidence that the death rate seems to be similar to, and not higher than, other patients with stable CAD (31, 32). In order to improve the quality of life in patients with refractory angina reduction in anginal symptoms is an important goal. It is essential to emphasize that among the choices for alternative options to offer to patients with refractory angina, the CSR should be considered, because it is a secure device and apparently effective in reducing anginal symptoms and in improving the quality of life in this category of patients (24). Expected data from ongoing studies among these patients will further evaluate the impact of CSR on angina symptoms and quality of life (https://ClinicalTrials.gov, NCT02710435/NCT01566175). Furthermore, future studies are needed to investigate and confirm the mechanism of action of the CSR with the use of more accurate imaging techniques such as the use of SPECT or fMRI.

## Author Contributions

The present work is not under consideration elsewhere, and all co-authors have had substantial scientific input and have agreed upon the current content.

## Conflict of Interest Statement

The authors declare that the research was conducted in the absence of any commercial or financial relationships that could be construed as a potential conflict of interest. Relationships with industry and financial associations that may create potential for conflict of interest are fully disclosed in the manuscript.
